# Alloy-Electrode-Assisted High-Performance Enhancement-Type Neodymium-Doped Indium-Zinc-Oxide Thin-Film Transistors on Polyimide Flexible Substrate

**DOI:** 10.34133/2021/5758435

**Published:** 2021-03-22

**Authors:** Kuankuan Lu, Rihui Yao, Wei Xu, Honglong Ning, Xu Zhang, Guanguang Zhang, Yilin Li, Jinyao Zhong, Yuexin Yang, Junbiao Peng

**Affiliations:** State Key Laboratory of Luminescent Materials and Devices, South China University of Technology, Guangzhou 510640, China

## Abstract

Flexible thin-film transistors with high current-driven capability are of great significance for the next-generation new display technology. The effect of a Cu-Cr-Zr (CCZ) copper alloy source/drain (S/D) electrode on flexible amorphous neodymium-doped indium-zinc-oxide thin-film transistors (NdIZO-TFTs) was investigated. Compared with pure copper (Cu) and aluminum (Al) S/D electrodes, the CCZ S/D electrode changes the TFT working mode from depletion mode to enhancement mode, which is ascribed to the alloy-assisted interface layer besides work function matching. X-ray photoelectron spectroscopy (XPS) depth profile analysis was conducted to examine the chemical states of the contact interface, and the result suggested that chromium (Cr) oxide and zirconium (Zr) oxide aggregate at the interface between the S/D electrode and the active layer, acting as a potential barrier against residual free electron carriers. The optimal NdIZO-TFT exhibited a desired performance with a saturation mobility (*μ*_sat_) of 40.3 cm^2^·V^−1^·s^−1^, an *I*_on_/*I*_off_ ratio of 1.24 × 10^8^, a subthreshold swing (SS) value of 0.12 V·decade^−1^, and a threshold voltage (*V*_th_) of 0.83 V. This work is anticipated to provide a novel approach to the realization of high-performance flexible NdIZO-TFTs working in enhancement mode.

## 1. Introduction

Today, Cu interconnection metallization, including bus lines and device electrodes, plays an important role in high refresh rate, ultra-high definition, and large size of flexible displays, owing to its low resistivity and good mechanical performance [1]. Although pure Cu has the advantages in terms of low resistivity [2, 3], it is difficult to be directly used as an electrode for a thin-film transistor (TFT) device due to its poor adhesion strength to a flexible substrate and copper atom diffusion issue. In view of this matter, copper alloying is a very promising copper metallization strategy without introducing a heterogeneous adhesion-barrier layer that leads to variety of etching problems in the subsequent photolithography process [4, 5]. Conventional binary Cu alloys, such as Cu-Ca [6], Cu-Mn [7, 8], and Cu-Ti [9], have been verified to act as electrodes in TFTs preliminarily. However, the resistivity is uncompetitive due to the relatively high content of doped metals. In addition, polymer materials are diffusely used in optoelectronic technology, such as flexible substrate, conductor, insulator, and packaging materials [10–12]. Flexible substrates such as polyethylene naphthalate (PEN), polyethylene terephthalate (PET), polycarbonate (PC), and polyimide (PI) are widely tried to be used in flexible electronics due to their advantages of transparency, light weight, flexibility, and sturdiness [13, 14]. Among them, PI is the most promising substrate for high-performance flexible display with Cu wiring because of its high thermal stability and good mechanical and chemical properties [15]. However, few studies specifically reported Cu alloy electrodes on PI substrates. As a result, low-resistivity Cu-Cr-Zr (CCZ) ternary alloy following the “microquantity and multielement” guideline was developed for a flexible PI substrate, which is proposed in our previous work [16].

Metal oxide materials have a wide range of electronical applications [17–20]. As an important component, amorphous metal oxide semiconductors (AOS) are developing rapidly in newly emerging application areas related to information display [21–24]. As a typical representative, indium- (In-) and zinc- (Zn-) based indium-gallium-zinc-oxide (IGZO) is a likely candidate to serve as the active layer of transistors in flexible display. Indeed, compared with conventional silicon- (Si-) based TFTs, AOSs exhibit attractive integrated electrical and optical properties such as good uniformity, high visible light transparency, high carrier mobility, low temperature preparation, and cost saving. The large spherical overlapping 5s orbitals from In is of profound significance for the carrier migration mechanism, leading to the insensitivity of the carrier transport to the amorphous phase. Zn plays an important role in the enhancement of amorphization and modulates the shallow tail state [25]. Ga doped in IZO, though deteriorating the mobility performance, suppresses the generation of oxygen-deficient defects (i.e., oxygen vacancies (*V*_O_) and metal interstitials), which improves the device stability [26]. As a matter of fact, a-IGZO has been commercialized. Nevertheless, considering that the high-resolution backplane or high-framerate 3D display requires a mobility of 20-50 cm^2^·V^−1^·s^−1^, some issues of AOS such as low mobility (IGZO ~10 cm^2^·V^−1^·s^−1^) and sensitivity to external environments are waiting to be resolved [27]. The insufficient mobility is due to the component of Ga, which is so high that degrades the mobility dramatically [28, 29]. In addition, Ga_2_O_3_ is acid-soluble and easy to be damaged in the wet etching process [30]. As a result, the carrier passivator, such as Ta, Zr, Si, and Hf, was added into the matrix (IZO) to improve the stability performance [31–34]. It is of particular interest to note that a microquantity dopant can also make an enormous influence on the device stability without significant mobility deterioration, since the electrical behavior of the active layer is forcefully dependent on the carrier passivator, which is ascribed to the differences of the bond-dissociation energy of the dopants [35]. Hence, the excessive carriers from *V*_O_ can be effectively alleviated by introducing more robust metal-oxygen bonds [36]. In comparison of all above carrier passivators, the rare earth element neodymium (Nd) is believed to be an excellent oxygen binder, which can suppress the generation of *V*_O_ and control the carrier concentration. In theory, lower work function metals such as Al, Ti, Mo, or Cu are preferred as S/D electrodes to the formation of ohmic contact. Nevertheless, except for electrode work function, it has been confirmed that different types of electrodes lead to different contact resistances and interface electrical characteristics, which depend on the electronic structure and carrier density of the channel contact surface [37]. As a result, it is possible to modulate the carrier transport through chemical reactions with a specially designed alloy electrode.

**Figure 1:**
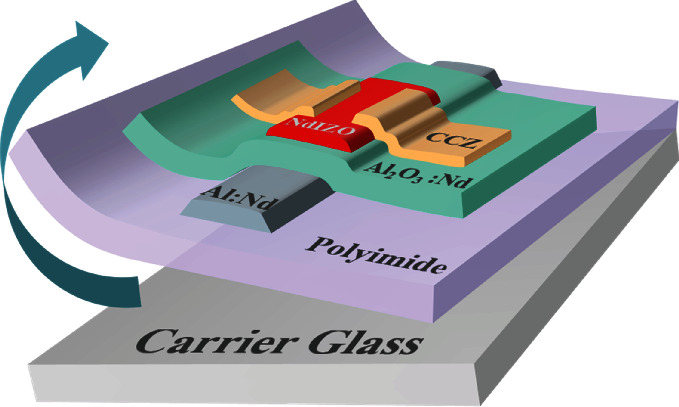
Structure diagram of a flexible thin-film transistor with CCZ S/D electrodes.

In this paper, the contact characteristics of the interface between the CCZ S/D electrode and NdIZO active layer were investigated. The typical structure of the device is shown in Figure 1. Different electrodes, i.e., CCZ, pure Cu, and pure Al, were utilized as the S/D electrode of the flexible NdIZO-TFTs, and the element diffusion as well as the chemical states was studied by XPS analysis. The work function match between different materials was demonstrated by Ultraviolet Photoelectron Spectrometer (UPS)/Ultraviolet-visible Spectrophotometer (UV-Vis) tests, which makes it possible to depict the energy band diagram of the carrier transport at the interface between different S/D electrodes and the NdIZO active layer.

## 2. Results and Discussion

### 2.1. The Electrical Performance of TFTs with Different S/D Electrodes

**Figure 2:**
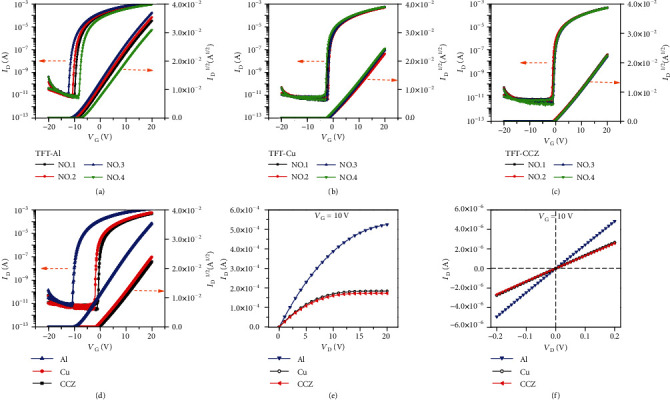
Electrical characteristics of NdIZO-TFTs: transfer curves with (a) Al S/D electrode, (b) Cu S/D electrode, and (c) CCZ S/D electrode. (d) Comparison of the three kinds of S/D electrodes; output curves of (e) the whole region with *V*_D_ from 0 V to 20 V and (f) the linear region with *V*_D_ from -0.2 V to 0.2 V.

Figure 2 shows the electrical characteristics of the NdIZO-TFTs with different S/D electrodes under optimal annealing temperature of 360°C, that is, the value of *I*_D_ (drain current) as a function of *V*_G_ (gate voltage) or *V*_D_ (drain voltage). As shown in Figures 2(a)–2(c), devices possess reliable reproducibility. Particularly, the NdIZO-TFTs with the CCZ S/D electrode (TFT-CCZ) have the best reproducibility, which reveals better stability than pure metals such as Cu and Al. And thanks to the high stability and adhesion strength of the CCZ electrode, the flexible device is so robust that it can withstand 120,000 (120k) times bending without obvious deterioration in transfer characteristics (Figure S1). Considering that the preparation conditions are completely identical, the difference of the transfer characteristics in Figure 2(d) can be ascribed to different S/D electrodes. It is interesting that TFT-CCZ shows positive threshold voltage (*V*_th_), which indicates enhancement working mode, while the NdIZO-TFTs with the pure Cu (TFT-Cu) or pure Al (TFT-Al) S/D electrode show negative threshold voltage (*V*_th_), which indicates depletion working mode. Both the TFT-Al and TFT-Cu exhibit normally on switching characteristic with a negative *V*_th_, which means residual free electron carrier transport from the source to drain electrode when without gate modulation voltage. The representative electrical parameters extracted from Figure 2(d) and parameters of NdIZO-TFT from other literatures are shown in Table 1 [38, 39]. On the whole, our work shows better performance due to reasonable process parameters than other literatures. It is found that the transfer characteristics of TFT-Cu and TFT-CCZ in Figure 1(d) exhibit parallel shift, resulting in the almost identical electrical parameters. While the mobility of TFT-Al is much higher than that of TFT-Cu and TFT-CCZ, the latter two exhibit improved SS and *V*_th_ values; moreover, the TFT-CCZ reveals further improvement of the threshold delay to an enhancement type. Since the only difference of the TFT devices is the S/D electrode material, the contact between the S/D electrode and the active layer plays a critical role in carrier injection.

The output characteristics of NdIZO-TFT with different electrodes are shown in Figure 2(e). The output current of TFT-Al is much higher than that of TFT-Cu and TFT-CCZ under the same gate voltage (*V*_G_) modulation, which reveals that the S/D electron injection for TFT-Al is much easier than that for the other two devices. There are two possible mechanisms to suppress the injection of electron: (i) potential barrier formed by the Schottky contact and (ii) electron block layer to reduce the conductivity of the contact region. Figure 2(f) shows the linear region *I*-*V* characteristics in the low-drain voltage regime under *V*_G_ = 10 V. The *I*-*V* linear relation without current crowding phenomenon indicates a totally ohmic contact between any kind of above electrodes and the NdIZO active layer.

### 2.2. The Interface Contact Characteristics between NdIZO and Different S/D Electrodes

**Figure 3:**
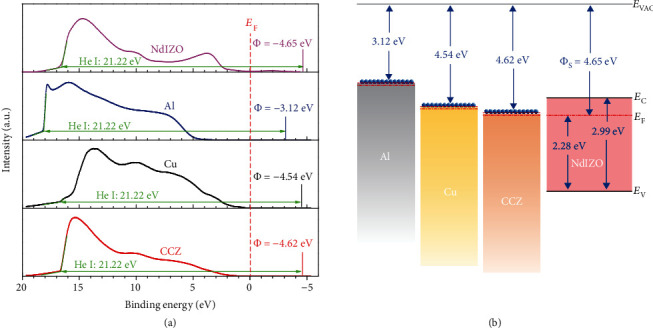
The work functions of the electrodes and active layer: (a) the UPS spectra of NdIZO, Al, Cu, and CCZ; (b) the schematic diagram of the energy level of the electrodes and active layer before contact.

The work functions of the electrodes were extracted from UPS spectra in Figure 3(a). As shown in the schematic diagram of the energy levels in Figure 3(b), the work functions of these three electrodes are all higher than the Fermi level of the active layer, corresponding to the ohmic contact demonstrated in Figure 3(b). In particular, the work function of CCZ is closest to the Fermi level of NdIZO, indicating a potential of good contact match with that of the active layer. Hence, the possibility of the Schottky contact rectification for TFT-Cu and TFT-CCZ is excluded.

**Figure 4:**
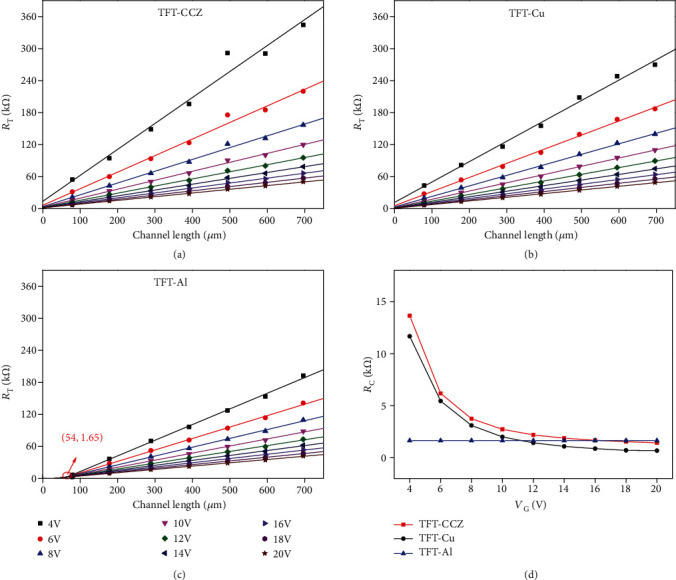
*R*_T_ as a function of channel length with different *V*_G_ for NdIZO-TFTs with (a) CCZ, (b) pure Cu, and (c) pure Al S/D electrodes. (d) Comparison of *R*_C_ for different S/D electrodes.

Although the ohmic contact of above electrodes is substantiated, there is no powerful evidence to prove that the electrical changes of the oxide semiconductor-based TFTs with different S/D materials are determined directly by work function of the S/D electrode, because of the high carrier concentration of the AOS [40]. Hence, the contact resistance (*R*_C_) with different S/D electrode materials was analyzed by using the transmission line model (TLM) [41], as shown in Figures 4(a)–4(c). The total resistance (*R*_T_) of the device is equal to the sum of the channel resistance (*r*_ch_*L*) and *R*_C_, where *R*_C_ can be expressed through *L*_T_ (the current transfer length), as shown in

(1) $$\begin{array}{c} R_{\text{T}}=\frac{V_{\text{D}}}{I_{\text{D}}}=r_{\text{ch}}L+R_{\text{C}}=r_{\text{ch}}L+r_{\text{ch}}\cdot 2L_{T}, \end{array}$$

where *r*_ch_ is the channel resistance per unit channel length (*L*). In the TLM plot, the horizontal coordinate of the cross point is equal to 2*L*_T_ and the vertical coordinate is equal to *R*_C_. In Figures 4(a) and 4(b), the fitted lines do not intersect at one point, and each intercept of the ordinate represents the *R*_C_ under a specific *V*_G_. In Figure 4(c), the fitted lines intersect at one point, resulting in constant *R*_C_, which is similar to the contact situation of Si-based TFTs with a highly doped ohmic (*n*^+^) region below the S/D electrodes. This similarity can be ascribed to the very low work function of Al. The comparison of *R*_C_ for different S/D electrodes is shown in Figure 4(d). The results show that both the *R*_C_ of TFT-CCZ and TFT-Cu will be modulated with the variation of *V*_G_: low *V*_G_ corresponding to high *R*_C_ and high *V*_G_ corresponding to low *R*_C_. Therefore, it can be speculated that the relatively high *R*_C_ of TFT-CCZ under low *V*_G_ is conducive to suppress the injection of electron in the subthreshold regime. When the *V*_G_ operates at high level, the *R*_C_ decreases rapidly, even smaller than the constant *R*_C_ of TFT-Al, thus insuring efficient injection of electron. It is believed that the modulation of *R*_C_ by *V*_G_ is the key to the realization of high-quality switching characteristic.

**Figure 5:**
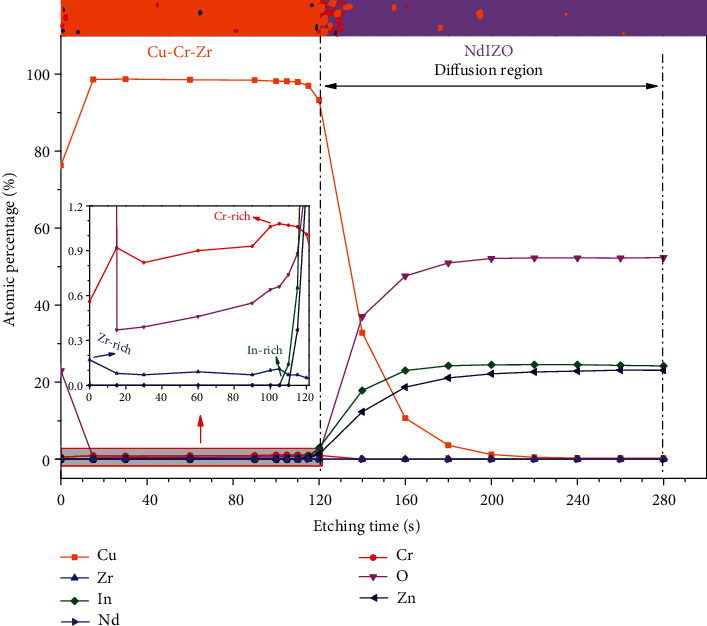
The element distribution of CCZ/NdIZO structure.

It is worth noting that the *R*_C_ of TFT-CCZ is always higher than that of TFT-Cu, which may be attributed to the interface reaction caused by the alloying of copper metal. To verify this conjecture, a double-layer sample of CCZ/NdIZO was prepared, and the thicknesses of the CCZ and NdIZO were 100 nm and 200 nm, respectively. The deposition and annealing parameters were identical with the TFT-CCZ device. XPS depth profile analysis of the sample was performed.  Figure 5 shows the element distribution by XPS depth profile analysis, and the inset shows the interface between the CCZ S/D electrode and NdIZO active layer. It can be seen that Zr and Cr, as refractory metals, do not form intermetallic compounds with Cu and segregate on the surface and interface, which is consistent with our previous work [42]. Moreover, this morphology acting as a self-aligned barrier buffer layer can effectively block the diffusion of Cu atoms into the NdIZO active layer, thereby preventing the activity of the NdIZO active layer from being affected by the Cu deep-level acceptor trap. In addition, the emergence of the In component before the Zn component indicates an In-rich region at the interface, which can be ascribed to the bombardment effect during CCZ deposition that breaks the weak In bond [43]. Besides, the steep profile of Cu in the NdIZO layer reveals an effective suppression of Cu atom diffusion by the interface segregation of Zr and Cr.

**Figure 6:**
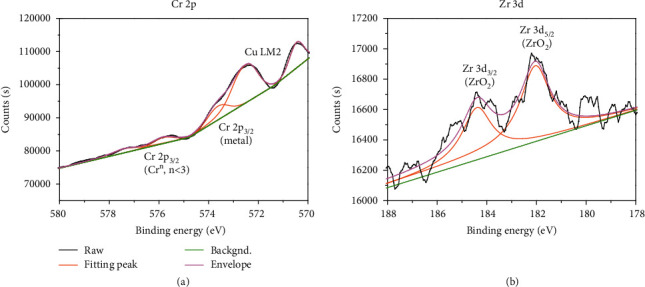
The XPS spectra for (a) Cr 2p and (b) Zr 3d at the interface of the CCZ/NdIZO sample.

To further investigate the chemical states of the Cr and Zr elements segregated at the contact interface, Cr 2p and Zr 3d spectra from the CCZ/NdIZO interface were analyzed. As shown in Figure 6(a), the Cr 2p peak can be fitted by three nearly Gaussian distributions, which are centered at 573.6 eV, 575.8 eV, and 577.3 eV. The three peaks are generally attributed to metal and suboxide. Figure 6(b) shows the Zr 3d peak fitted by two nearly Gaussian distributions, which are centered at 182 eV (Zr 3d_5/2_) and 184.4 eV (Zr 3d_3/2_). The Zr 3d peaks determine that Zr exists in the form of oxide (ZrO_2_) at the interface. As a result, an oxide layer from Cr and Zr is confirmed at the contact interface, which accounts for higher *R*_C_ of TFT-CCZ.

**Figure 7:**
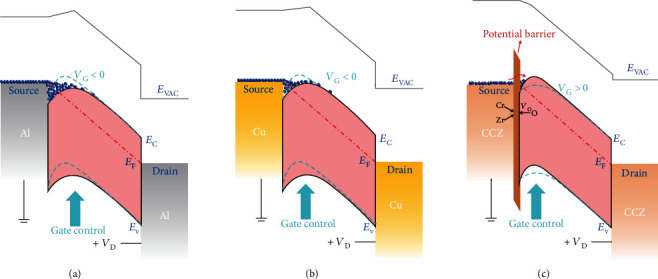
Schematic diagram of the energy band for carrier transportation from source to drain: (a) TFT-Al, (b) TFT-Cu, and (c) TFT-CCZ.

On the basis of the above analyses and results, a hypothetic energy band diagram of carrier transport between the source and drain is proposed to explain why different S/D electrodes will affect carrier injection dramatically. As shown in Figure 7(a), due to the very low work function of the Al electrode, the end of the energy band will bend significantly and form potential well through the metal-semiconductor contact effect. Thus, a very negative gate voltage is required to deplete the residual free electrons, which is equivalent to increasing the potential barrier of the conduction band. A Cu electrode with a higher work function will improve this situation, as shown in Figure 7(b). However, the channel still cannot be pinched off without a negative gate voltage due to the high conductivity of NdIZO that ensures high mobility. As a consequence, a self-assembled oxide block layer is introduced on the contact region with the assistance of the CCZ alloy electrode, as shown in Figure 7(c). The carriers need to tunnel through the additional block layer, which is equivalent to increasing the potential barrier of the conduction band. Therefore, an enhancement mode with a normally off channel is realized since a positive gate voltage is needed to turn on the channel.

## 3. Conclusion

High-performance enhancement-type flexible NdIZO-TFTs were realized by using the CCZ alloy electrode. The influence of S/D electrodes on the electrical performance of NdIZO-TFT was investigated. The *R*_C_ of TFT-CCZ is modulatable by gate voltage. Compared with Al and Cu electrode materials, the CCZ electrode will increase the *R*_C_ by the self-assembled oxide layer at the contact region, which acts as a potential barrier to suppress the residual free electron injection and ensure enhancement mode without deteriorating the electrical performance significantly. The self-assembled auxiliary interface layer that blocks residual free electron makes it possible to fabricate high-performance TFTs using more conductive semiconductor which provides a novel approach to the realization of high-performance flexible NdIZO-TFTs working in enhancement mode.

## 4. Experimental Details

### 4.1. Materials and Methods

As delineated in Figure 1, the NdIZO-TFT was manufactured on 18 *μ*m PI film supported by ultraflat carrier glass. The gate electrode (GE)/gate insulator (GI) was prepared by using a 300 nm Al-Nd alloy film that partly anodized into 200 nm AlO*_x_*:Nd GI, as described in literatures [44, 45]. Then, a 35 nm active layer was deposited at room temperature through a stencil shadow mask by radiofrequency magnetron sputtering. The specific composition of the NdIZO target is Nd_2_O_3_ : In_2_O_3_ : ZnO = 1 : 62.5 : 36.5 wt.%. The film deposition was carried out in Ar/O_2_ (100 : 1) atmosphere at a deposition pressure of 3 mTorr and a sputtering power of 60 W (i.e., 3 W·cm^−2^). Afterwards, the device was annealed in air atmosphere at 360°C for 1 h. In the end, a 150 nm CCZ film was deposited to act as S/D electrodes (*L*/*W* = 360 *μ*m/540 *μ*m). The same fabrication process was applied to compare devices except for the S/D electrode metal materials (Al and Cu).

### 4.2. Analytical Methods

The electrical characteristics of TFT devices were measured by using a semiconductor analyzer (Agilent 4155C) in dark and air environment. The chemical changes of the channel region were detected by X-ray photoelectron spectroscopy (XPS) measurements, and the work function was detected by ultraviolet photoelectron spectroscopy (UPS) measurements (ESCALAB250Xi, Thermo Fisher Scientific, Waltham, MA, USA) at a basic pressure of 7.5 × 10^−10^ Torr. The optical band gap was detected by using an ultraviolet spectrophotometer (SHIMADZU UV2600, SHIMADZU, Tokyo, Japan). The *R*_T_ lines were fitted by means of partial least squares regression by Origin 9 software.

## Figures and Tables

**Table 1:** Electrical parameters of above devices annealed at 360°C and parameters of NdIZO-TFT from other literatures.

S/D electrode	*I*_ON_/*I*_OFF_	*μ*_sat_ (cm^2^·V^−1^·s^−1^)	SS (V·dec^−1^)	*V*_th_ (V)	Notes
Al	1.93 × 10^8^	52.6	0.23	-8.5	This work
Cu	1.79 × 10^8^	40.8	0.10	-1.1	This work
CCZ	1.24 × 10^8^	40.3	0.12	0.83	This work
ITO	~10^7^	30.4	0.26	-4.74	Ref. [38]
ITO	~10^6^	4.25	0.34	-0.97	Ref. [39]

## Data Availability

The data used to support the findings of this study are included within the article and the supplementary materials.

## Supplementary Materials

Supplementary Materials Figure S1: dynamic bending test: (a) principle of the bending machine; (b) transfer characteristics of TFT-CCZ with dynamic bending up to 120k times.
