# Supplementary material for: Alloy-Electrode-Assisted High-Performance Enhancement-Type Neodymium-Doped Indium-Zinc-Oxide Thin-Film Transistors on Polyimide Flexible Substrate
Source: Research (Wash D C). 2021 Mar 22;2021:5758435. doi: 10.34133/2021/5758435 (PMC8010622; doi:10.34133/2021/5758435)
Supplement: Supplementary Materials — Figure S1: dynamic bending test: (a) principle of the bending machine; (b) transfer characteristics of TFT-CCZ with dynamic bending up to 120k times. [file 5758435.f1.docx]

Alloy-electrode-assisted High-performance Enhancement-type Neodymium Doped Indium-zinc-oxide Thin Film Transistors on Polyimide Flexible substrate

Kuankuan Lu^1^, Rihui Yao^1^, Wei Xu^1^, Honglong Ning^1^, Xu Zhang^1^, Guanguang Zhang^1^, Yilin Li^1^, Jinyao Zhong^1^, Yuexin Yang^1^ and Junbiao Peng^1^

1 State Key Laboratory of Luminescent Materials and Devices, South China University of Technology, Guangzhou 510640, China

Correspondence should be addressed to Honglong Ning; ninghl@scut.edu.cn and Junbiao Peng; psjbpeng@scut.edu.cn


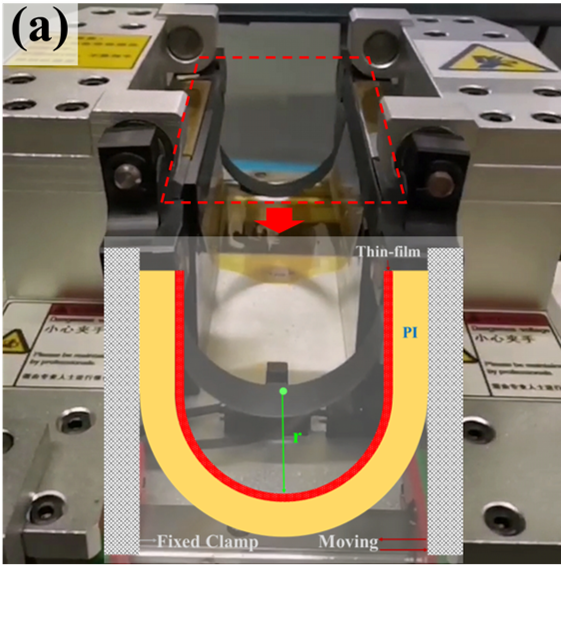


**Figure S1.** Dynamic bending test: (a) Principle of the bending machine; (b) transfer characteristics of TFT-CCZ with dynamic bending up to 120k times
